# Updating professional development for medical librarians to improve our evidence-based medicine and information literacy instruction

**DOI:** 10.5195/jmla.2018.386

**Published:** 2018-07-01

**Authors:** Joseph Costello

**Affiliations:** Informationist, Homer Stryker M.D. School of Medicine, Western Michigan University, Kalamazoo, MI

## Abstract

Medical librarians lack professional development opportunities in the critical appraisal of biomedical evidence. An update to our professional development opportunities could support our efforts to teach critical appraisal of biomedical evidence during evidence-based medicine or information literacy instruction. If we enhance our understanding of latent influences on evidence quality—such as changes to Food and Drug Administration regulations, predatory or deceptive publishing practices, and clinical trial study designs—we can improve our value to medical education and hospital systems.

Understanding health care in the United States requires a sustained effort, as it is always changing. As the US health care system undergoes changes, whether it be a new law or medical treatment breakthrough, it behooves medical librarians to understand the process by which these changes occur. New policies and legislation, such as the 21st Century Cures Act (Cures Act), open up opportunities for medical librarians to develop a richer knowledgebase. Physician groups forming to combat medical distrust or unnecessary medical recommendations (e.g., American Board of Internal Medicine’s Choosing Wisely initiative, Right Care Alliance) indicate the need for trusted analysis of biomedical literature. If we enhance our understanding of the step-by-step processes necessary to implement changes in the health care system, we can better predict the emergence of user information needs (e.g., a patron who hopes to enhance their ability to appraise a landmark study or is curious about how a controversial treatment gains government approval).

To further appreciate the information needs of our patrons and improve our delivery of biomedical information and education, we could benefit from an expansion in our professional development offerings. I propose that we introduce content into our professional development opportunities about the interplay between such areas as medical education curriculum design, clinical trial study designs, the history of Food and Drug Administration (FDA) policies (e.g., medical therapy or device approval processes and reversals), and scholarly publishing (e.g., predatory or deceptive publishing practices and conflicts of interest). These areas all play an integral part in constructing medical recommendations. With a better understanding of these influences that bear upon the evidence leading to medical recommendations, we could update our knowledgebase and deliver more interesting, pragmatic evidence-based medicine (EBM) or information literacy instructional sessions.

When and how we introduce the above topics are important considerations. During medical school, academic medical librarians face the challenge of overcoming relevancy and context gaps when delivering EBM content [1]. For medical students in the foundational science years of their education (i.e., the first one or two years of traditional medical school curriculum), showcasing an information resource using a patient, intervention, comparison, outcome (PICO)–based clinical scenario lacks *relevance* to their current curriculum and testing schedule. Without being engaged in clinical experiences, students lack adequate understanding of the clinical *context* in which to place the material that we deliver.

However, some medical schools are shifting to a more integrated curriculum, in which basic science and clinical science are taught together in all three to four years. If this trend continues, our algorithm of a clinical scenario—question → PICO → search → retrieve → basic appraisal—may allay relevancy and context gaps, allowing us to focus more fully upon appraisal instruction. Advancing our appraisal instruction is an opportunity to place the biomedical literature into such a context where we elucidate some of the influences (e.g., clinical trial study designs, FDA policies) that weigh upon the quality of the evidence.

To help our learners build an understanding of these influences on the evidence obtained through these information resources, we have to have a better understanding of the relationships among clinical trials, regulatory policies, and scholarly publishing. Imagine an update to our delivery of EBM or information literacy sessions, where we progress through more nuanced discussions about the fact that a surrogate end point “that is not itself a direct measurement of clinical benefit” can be used as proof-of-concept for a therapy and that it is possible that evidence of benefit for “breakthrough therapies” now “means data…other than randomized clinical trials” [2]. We could augment our discussions about the evidence pyramid or critical appraisal by showing how these changes can weaken standards for new therapies.

Or imagine sessions where rather than simply showing students how to search for evidence related to mammography screening outcomes, we introduce some conflicting peer-reviewed recommendations leading to the controversy surrounding appropriate mammography screening ages and ask learners to debate this controversy by further identifying the literature that supports either side. We may suddenly improve how engaging our sessions are for learners, whether they are in science or medicine curricula, because we place learners in positions where they can leverage more of their natural critical thinking strengths to engage the material and the clinical context. We allow learners not only to search and appraise evidence, but also to engage with the material and participate in conversations around the material. These types of exercises and discussions would help deliver more comprehensive EBM *and* information literacy education. In other words, they help support a type of belongingness to and immersion into health care culture.

If academic medical librarians do open EBM sessions by sharing how low-quality evidence can lead to poor or tragic patient outcomes (e.g., Willman [3]), we will alert our learners as to why it is important to strengthen their information literacy skills to prevent these errors from recurring. How learners can eventually prevent medical errors requires, in part, an advanced understanding of clinical trial study design, the FDA, and scholarly publishing practices. Poor study design can lead to significant findings that are false [4]. The FDA no longer requires improved patient outcomes as a metric of quality. Furthermore, in addition to predatory and deceptive publishing practices, clinical experts who edit content on pre-appraised evidence platforms, as well as guideline sponsors and authors, sometimes have conflicts of interest [5, 6]. Collectively, curriculum issues, trial designs, regulatory policies, and scholarly publishing impact evidence quality and, hence, influence medical recommendations. We could introduce EBM or information literacy sessions with updates on the political and industrial systems of education, research, scholarly publishing, and health care policy-making.

In private practice, providers are met with challenges to their information literacy (e.g., pharmaceutical reps, curious patients). We need to ensure that we prepare medical learners to think critically about evidence that is presented to them. Medical librarians in the nonacademic health care setting can reinforce what we introduce in medical school by illustrating to clinical teams that there are many influences that have the potential to undermine a medical recommendation and that we are here to help ensure that they receive the information support to ensure they are making informed medical decisions. In essence, we are augmenting information literacy training and increasing our value to the organization in a timely manner as hospital librarians face shifts in staffing (e.g., Schwartz and Elkin [7]). Coupled with the increasing popularity of costly pre-appraised evidence platforms (e.g., DynaMed, UpToDate) where bias is present [5], risks to a physician’s information literacy skillset may increase.

To illustrate, let me offer an example of how aforementioned changes in my own knowledgebase have influenced my practice. In December 2016, Congress passed the Cures Act. The FDA states that the Cures Act will “accelerate” the introduction of new therapy options to the market, as there are “patients who need them faster and more efficiently” [8]. The Cures Act occurs at a time when some of the current, most costly medical recommendations already lack evidence of improved patient outcomes (e.g., Al-Lamee et al. [9]).

I find it humbling that my awareness of the Cures Act came via an article in *The Atlantic* about the trend of physicians making medical recommendations based on poor evidence [10]. This article was shared with me by a third-year medical student after I spoke with him about a new textbook that introduced me to the phenomenon of medical reversals [11]. In *The Atlantic* article, I read that a candidate for head of the FDA had gone on the record saying that we should “let people start using them [treatments] at their own risk” [10]. The FDA leadership candidate’s perspective is not entirely novel, I discovered. There is a pattern of acceptance of lower evidence as a basis for medical recommendations (e.g., Willman, Ioannidis, and Prasad et al. [3, 12, 13]).

What is novel now is that the Cures Act seems to signal an acceptance of medical recommendations with lower evidence standards that do not produce improvements in patient outcomes. Anecdotally, when I asked some faculty colleagues about the Cures Act, no one had heard of it. This has led to many discussions with faculty on the range of medical recommendations with dubious evidence to support them. In the classroom, only one student had heard of the Cures Act but did not know any details.

Therefore, I introduced the Cures Act, medical recommendations with dubious evidence, and medical reversals in an introductory EBM session and witnessed a significant increase in student interest. As a result, librarians at our institution continue to update learners, staff, and faculty on predatory publishing (e.g., PubMed’s “backdoor,” vetting publisher’s email solicitations) and work diligently to discuss the conflicts of interest that can occur on trusted pre-appraised evidence platforms or in guideline authorship. Our learners’ overall interest in gaining a better understanding of how medical recommendations are constructed has extended to residents and some expert clinicians, and has generated more education sessions led by librarians on these topics.

We do not want a generation of physicians practicing medicine without understanding how the evidence on which they are basing their decisions may misrepresent true clinical benefit to patients. Training students on the minutia of the human body (e.g., enzymes, molecules, pathways), as occurs in medical school, can introduce reductionist thinking, which reduces complex patient scenarios to a single molecule or pathway that causes illness. Similarly, in our practice as medical librarians, we often ask learners to reduce a complex scenario to a single question and further into a simple search.

Although we already offer some basic appraisal strategies, I am arguing that an update to our knowledgebase can help us better empower our learners. Holistic learning about the health care system and scholarly culture that I am proposing—for example, knowing that the passage of the Cures Act further reduced funding for preventive medicine and allowed lower evidence thresholds for new therapies to be signed into law—can help medical librarians better adjust to information literacy challenges that health care professionals face.

With this knowledge, we may be better able to anticipate information requests from our patrons or at least augment our current delivery of EBM or information literacy sessions. We have an opportunity to showcase information resources with a more precise framework that could help learners better assess the benefits and flaws of the informational materials that we teach them how to access. While complications in the US health care system are myriad, how we frame these complex challenges through the lens of industry, government, and evidence standards in classrooms or clinic settings may help allay anxieties that can arise due to limitations that are present in sources of evidence. We should encourage learners’ natural critical thinking strengths and offer a more substantial supportive role to help them reinforce their ability to see more sides of the ever-changing story of the health care system.

## References

[1] Maggio LA, ten Cate O, Chen HC, Irby DM, O’Brien BC (2015). Challenges to learning evidence-based medicine and educational approaches to meet these challenges. Acad Med.

[2] (2015). 21st Century Cures Act. Public law 114–255 (H.R. 34) 114th Congress.

[3] Willman D (2000). POSICOR: 143 sudden deaths did not stop approval. Los Angeles Times.

[4] Ioannidis JPA (2005). Why most published research findings are false. PLOS Med [Internet].

[5] Amber KT, Dhiman G, Goodman KW (2014). Conflict of interest in online point-of-care clinical support websites. J Med Ethics.

[6] Sox HC (2017). Conflict of interest in practice guidelines panels. JAMA.

[7] Schwartz DG, Elkin PL (2017). Health sciences library closings: a context sensitive pilot study. Stud Health Technol Inform.

[8] US Food and Drug Administration 21st Century Cures Act [Internet].

[9] Al-Lamee R, Thompson D, Dehbi HM, Sen S, Tang K, Davies J, Keeble T, Mielewczik M, Kaprielian R, Malik IS, Nijjer SS, Petraco R, Cook C, Ahmad Y, Howard J, Baker C, Sharp A, Gerber R, Talwar S, Assomull R, Mayet J, Wensel R, Collier D, Shun-Shin M, Thom SA, Davies JE, Francis DP, ORBITA investigators (2018). Percutaneous coronary intervention in stable angina (ORBITA): a double-blind, randomised controlled trial. Lancet.

[10] Epstein D, Propublica (2017). When evidence says no, but doctors say yes. The Atlantic [Internet].

[11] Prasad V, Cifu A (2015). Ending medical reversal: improving outcomes, saving lives.

[12] Ioannidis JP (2008). Effectiveness of antidepressants: an evidence myth constructed from a thousand randomized trials?. Philos Ethics Humanit Med.

[13] Prasad V, Vandross A, Toomey C, Cheung M, Rho J, Quinn S, Chacko SJ, Borkar D, Gall V, Selvaraj S, Ho N, Cifu A (2013). A decade of reversal: an analysis of 146 contradicted medical practices. Mayo Clin Proc.

